# Peace of mind and anxiety in the waking state are related to the affective content of dreams

**DOI:** 10.1038/s41598-018-30721-1

**Published:** 2018-08-24

**Authors:** Pilleriin Sikka, Henri Pesonen, Antti Revonsuo

**Affiliations:** 1 Department of Psychology and Speech-Language Pathology, Turku Brain and Mind Center, University of Turku, Turku, Finland; 2 Department of Cognitive Neuroscience and Philosophy, School of Bioscience, University of Skövde, Skövde, Sweden; 3 Department of Mathematics and Statistics, University of Turku, Turku, Finland; 4 Department of Computer Science, Aalto University, Helsinki, Finland

## Abstract

Waking mental well-being is assumed to be tightly linked to sleep and the affective content of dreams. However, empirical research is scant and has mostly focused on ill-being by studying the dreams of people with psychopathology. We explored the relationship between waking well-being and dream affect by measuring not only symptoms of ill-being but also different types and components of well-being. Importantly, this is the first time peace of mind was investigated as a distinct aspect of well-being in a Western sample and in relation to dream content. Healthy participants completed a well-being questionnaire, followed by a three-week daily dream diary and ratings of dream affect. Multilevel analyses showed that peace of mind was related to positive dream affect, whereas symptoms of anxiety were related to negative dream affect. Moreover, waking measures were better related to affect expressed in dream reports rather than participants’ self-ratings of dream affect. We propose that whereas anxiety may reflect affect dysregulation in waking and dreaming, peace of mind reflects enhanced affect regulation in both states of consciousness. Therefore, dream reports may possibly serve as markers of mental health. Finally, our study shows that peace of mind complements existing conceptualizations and measures of well-being.

## Introduction

Although it is increasingly recognized that sleep is closely linked to waking mental well-being^1–4^, the relationship between dreams (or dreaming) and well-being is still largely unknown. Dreams are the subjective experiences that occur during sleep and dreaming is the process of having such experiences. Because dream experiences cannot be directly accessed, it is only possible to study dreams as they are recalled and reported in the waking state (i.e., *data* in the form of narrative dream reports or ratings of different aspects of the dream experience are collected to study the *phenomena* of dreams and dreaming). In clinical practice dream experiences have long been considered markers of mental health^5^. Several current theories assume that dream experiences, especially affective experiences, not only reflect^6,7^ but are also influenced by^8,9^ and themselves influence waking well-being^10–14^. However, empirical research has mostly focused on ill-being by studying the dreams of people with symptoms of psychopathology. But well-being is not just the absence of symptoms of ill-being^15,16^ and therefore we cannot draw conclusions regarding well-being by studying psychopathology. Scientific research on well-being has evolved rapidly and currently several different types and components of well-being are distinguished. Only a few studies have explored the relationship between well-being, as it is conceptualized in the science of well-being, and dream content. Moreover, Eastern conceptualizations of well-being, such as peace of mind^17^, are often overlooked in well-being research and not addressed in dream research, even though these may constitute an important aspect of well-being also in Western cultures. Here we integrate the two different fields of research — well-being and dream research — and explore the relationship between waking well-being and dream affect by measuring not only the symptoms of ill-being but well-being as conceptualized in the science of well-being. Moreover, this is the first study to investigate peace of mind in a Western sample as part of a comprehensive framework of well-being and in relation to dream content.

Well-being is a complex multifaceted construct. In the science of well-being two different types of well-being are distinguished — hedonic and eudaimonic well-being^18^ cf.^19,20^. The concept of hedonic well-being (HWB) is typically operationalized as *subjective well-being*^21,22^ consisting of two affective components (positive affect, negative affect) and two cognitive components (life satisfaction, domain satisfaction)^23,24^. Thus, well-being in this conceptualization means experiencing high levels of positive affect, low levels of negative affect, and evaluating oneself to be satisfied with one’s life as a whole as well as with different life domains (e.g., relationships, work, health). The concept of eudaimonic well-being (EWB) refers to optimal functioning, a pursuit for excellence that contributes to personal growth and fulfilment of one’s potential as a human being^25,26^. The conceptualizations and operationalizations of EWB vary considerably, but having meaning and purpose in life are central to almost all the different approaches^25,27^.

These conceptualizations of well-being have mostly been developed in Western cultures. Eastern philosophy (i.e., Confucianism, Buddhism, Hinduism, and Taoism) and recent empirical research conducted in Eastern countries suggest that inner peace and harmony are central to well-being^17,28–30^. Lee *et al*.^17^ developed the construct peace of mind (PoM) and the Peace of Mind Scale (PoMS) to measure this form of well-being in the Chinese culture. The authors found PoMS to be positively correlated with measures of life satisfaction and positive affect, and negatively correlated with measures of negative affect, depression, and anxiety. PoMS also predicted measures of ill-being (i.e., anxiety, depression) beyond that of conventional HWB measures. The authors liken PoM to low-arousal positive affect but argue that it is nevertheless distinct from and reflects “a more complex and balanced state of mind” (p. 587) than low-arousal positive affect. Although individuals in Western cultures also associate well-being with inner peace and harmony^31–33^, there has been little effort in integrating those approaches into well-being research^34,35^. PoM as such has not been studied as part of a comprehensive framework of well-being in Western societies.

In clinical populations, there is considerable evidence that waking ill-being is related to dream affect (here the term affect is used to refer to the subjective experience of emotions and moods, i.e., feelings^36^). People with different mental health disorders (e.g., anxiety, depression), sleep disorders, and health behavior problems report more nightmares^8,37,38^ and negatively toned dreams in general^39^. Interestingly, the reduction of depressive symptoms as a result of antidepressant treatment has been shown to accompany a corresponding change in dream affect^40,41^. However, a large part of the evidence derives from epidemiological or questionnaire-based studies (rather than studies in which people have been asked to rate or report their dream experiences immediately upon awakening). The validity of results obtained with such methods can be questioned because what has been measured are people’s cognitive evaluations or beliefs about their dream experiences rather than the actual (recalled and reported) dream experiences^42,43^.

In non-clinical populations, research on the relationship between waking ill-being and dream affect is scarce and inconsistent. Some studies have found that individuals with anxious and depressive mood states rate their dreams as more unpleasant, report more frequent unpleasant dreams^44^ and express more negative affect in their dream reports^45^. In line with these findings, it has also been shown that individuals with higher scores of trait anxiety and neuroticism as well as with symptoms of general psychopathology express more negative affect in their dream reports^46^. Other studies have failed to demonstrate significant relationships between dream affect and anxiety, symptoms of general psychopathology or depression^47–50^.

The relationship between waking well-being and dream affect has been explored in only a few studies^51–53^. These studies have only measured HWB or some of its components (positive and negative affect and/or life satisfaction). Moreover, results of those studies are contradictory. For example, whereas Gilchrist *et al*.^51^ reported a negative relationship between life satisfaction and the ratings of negative dream affect, St-Onge *et al*.^53^ failed to detect any significant relationships between life satisfaction and the ratings of (positive or negative) affect in dreams. Additionally, whereas waking state affect (i.e., daily mood) has been found to correspond to respective dream affect (i.e., the more positive pre-sleep mood the more positive dream affect)^51,52,54^, waking trait positive and negative affectivity has not been found to be correlated with dream affect^51^. So far, no studies have investigated how EWB or PoM are related to dream affect.

In sum, in non-clinical populations research on the relationship between dream affect and waking ill- and well-being is limited and results inconsistent. Moreover, the different aspects of ill-and well-being have not been studied within a single framework. Therefore, in the present study we investigated the relationship between waking well-being and dream affect in a comprehensive framework by including the measures of both ill-being (i.e., symptoms of depression and anxiety) and well-being (i.e., life satisfaction, domain satisfaction, positive affect, negative affect, eudaimonic well-being, peace of mind). Specifically, our objective was to explore which aspects of waking ill-being and well-being are related to the affective content of subsequent dreams. Due to the scarcity and conflicting nature of research on the topic, this study was mostly exploratory in nature. However, based on existing studies we expected higher levels of waking well-being to be associated with more positive and less negative ratings of dream affect, and higher levels of waking ill-being to be associated with more negative and less positive ratings of dreams affect.

Healthy participants completed a well-being questionnaire, followed by a daily dream diary during the subsequent three-week period (i.e., 21 days) in which they reported all their dreams and rated the affect they experienced in the preceding dream using the modified Differential Emotions Scale^55^. In addition to self-ratings of dream affect, written dream reports were content analysed by two independent judges using the same scale (referred to as external ratings). We used both self- and external ratings of dream affect because previous studies have used either one or the other measurement method and this may be one reason for existing discrepancies in findings^56–59^. Because the daily ratings of dream affect (*N* = 552, *M* = 12.55, *SD* = 5.72) were nested within individuals (*N* = 44), multilevel regression models were performed in which the waking well-being and ill-being measures were used to predict self-rated and externally rated dream affect.

## Results

### Descriptive Statistics of Well-Being Measures

Table 1 summarizes the descriptive statistics of the unstandardized mean or sum scores for each well-being scale. The scores obtained in the current sample correspond well to the established norms or mean scores published in literature for healthy younger adults. As such, the findings are well in line with previous studies.

**Table 1:** Reliability and Descriptive Statistics of Well-Being and Ill-Being Measures.

	Type of Well-Being	Component of Well-Being	Scale	*α*	*M*	*SD*	*Mdn*	Actual min-max	Possible min-max
Mental Well-Being	Hedonic Well-being	Life satisfaction	Satisfaction With Life Scale (SWLS)	0.82	25.48	5.38	26.00	11–34	5–35
		Domain satisfaction	Brunnsviken Brief Quality of Life Scale (BBQ)	0.77^a^	62.45	19.42	61.00	4–92	0–96
		Positive affect	PANAS_PA	0.91	29.23	9.30	28.00	12–47	10–50
			mDES_PA	0.91	2.26	0.92	2.30	0.20–3.80	0–4
			12-PAC_HPA	0.91	2.57	1.14	2.50	1–5	1–5
			12-PAC_LPA	0.92	2.84	1.09	3.00	1–5	1–5
		Negative affect	PANAS_NA	0.86	16.86	6.77	14.50	10–35	10–50
			mDES_NA	0.87	0.78	0.73	0.60	0.00–2.80	0–4
			12-PAC_HNA	0.74	1.69	0.79	1.50	1–4	1–5
			12-PAC_LNA	0.67	2.45	0.87	2.50	1–4	1–5
	Eudaimonic Well-Being		Flourishing Scale (FS)	0.93	44.64	9.01	46.00	11–56	8–56
	Peace of Mind		Peace of Mind Scale (PoMS)	0.90 (0.86)	2.99 (3.27)	0.86 (0.76)	3.00 (3.29)	1.20–5.00 (1.43–5.00)	1–5
Mental Ill-Being	Depression		Depression scale from Patient Health Questionnaire (PHQ-9)	0.81	5.64	4.46	5.50	0–17	0–27
	Anxiety		Generalized Anxiety Disorder Scale (GAD-7)	0.91	4.48	4.92	3.00	0–20	0–21

*Note*. *N* = 44. ^a^Based on the six product scores (i.e., by multiplying the satisfaction and importance ratings for each domain of life). Values in parentheses indicate 7-item PoMS scores. PANAS_PA = positive affect subscale of the Positive and Negative Affect Schedule; mDES_PA = positive affect subscale of the modified Differential Emotions Scale; 12-PAC_HPA = high-arousal positive affect subscale of the 12-Point Affect Circumplex Scales; 12-PAC_LPA = low-arousal positive affect subscale of the 12-Point Affect Circumplex Scales; PANAS_NA = negative affect subscale of the Positive and Negative Affect Schedule; mDES_NA = negative affect subscale of the modified Differential Emotions Scale; 12-PAC_HNA = high-arousal negative affect subscale of the 12-Point Affect Circumplex Scales; 12-PAC_LNA = low-arousal negative affect subscale of the 12-Point Affect Circumplex Scales.

### Intercorrelations Between Well-Being Measures

Table 2 presents zero-order Spearman correlations between the well-being scales. For ease of interpretation these were computed using the unstandardized sum or mean scores of well-being scales. All the correlations were in the expected direction. The highest correlations (>0.8) occurred between the scales measuring the same underlying construct, i.e., positive and/or negative affect. Because of the latter, for subsequent analyses a composite measure of waking positive affect (PA) and negative affect (NA) was created (see Statistical Analyses for a detailed description). As can be seen from the table, the Peace of Mind Scale (PoMS) was not significantly correlated with low-arousal positive affect (12-PAC_LPA) which indicates that these two aspects of well-being are distinct constructs.

**Table 2:** Zero-order Correlations Between Well-Being Measures (*N* = 44).

	SWLS	BBQ	PANAS_PA	PANAS_NA	mDES_PA	mDES_NA	12-PAC_HPA	12-PAC_LPA	12-PAC_HNA	12-PAC_LNA	FS	PoMS	PHQ-9
SWLS													
BBQ	0.627**												
PANAS_PA	0.412**	0.251											
PANAS_NA	−0.560**	−0.324*	−0.315*										
mDES_PA	0.503**	0.297*	0.797**	−0.467**									
mDES_NA	−0.401**	−0.207	−0.273	0.827**	−0.364*								
12-PAC_HPA	0.419**	0.211	0.831**	−0.421**	0.753**	−0.324*							
12-PAC_LPA	0.331*	0.202	0.620**	−0.570**	0.669**	−0.541**	0.621**						
12-PAC_HNA	−0.345*	−0.128	−0.265	0.804**	−0.338*	0.835**	−0.300*	−0.506**					
12-PAC_LNA	−0.329*	−0.084	−0.460**	0.521**	−0.433**	0.568**	−0.467**	−0.574**	0.590**				
FS	0.634**	0.716**	0.371*	−0.429**	0.515**	−0.344*	0.383*	0.386**	−0.206	−0.276			
PoMS	0.590**	0.590**	0.386**	−0.355*	0.357**	−0.380*	0.286	0.262	−0.214	−0.325*	0.634*		
	(0.612**)	(0.544**)	(0.401**)	(−0.379*)	(0.422**)	(−0.393*)	(0.314*)	(0.319*)	(−0.243)	(−0.276)	(0.568**)		
PHQ-9	−0.535**	−0.422**	−0.440**	0.600**	−0.531**	0.581**	−0.496**	−0.467**	0.482**	0.450**	−0.492**	−0.581**	
												(−0.581**)	
GAD-7	−0.396**	−0.269	−0.423**	0.655**	−0.524**	0.673**	−0.392**	−0.532**	0.636**	0.567**	−0.276	−0.428**	0.668**
												(−0.433**)	

*Note*. Spearman correlations (two-tailed). Values in parentheses indicate correlation coefficients for the 7-item PoMS. SWLS = Satisfaction With Life Scale; BBQ = Brunnsviken Brief Quality of Life Scale; PANAS_PA = positive affect subscale of the Positive and Negative Affect Schedule; PANAS_NA = negative affect subscale of the Positive and Negative Affect Schedule; mDES_PA = positive affect subscale of the modified Differential Emotions Scale; mDES_NA = negative affect subscale of the modified Differential Emotions Scale; 12-PAC_HPA = high-arousal positive affect subscale of the 12-Point Affect Circumplex Scales; 12-PAC_LPA = low-arousal positive affect subscale of the 12-Point Affect Circumplex Scales; 12-PAC_HNA = high-arousal negative affect subscale of the 12-Point Affect Circumplex Scales; 12-PAC_LNA = low-arousal negative affect subscale of the 12-Point Affect Circumplex Scales; FS = Flourishing Scale; PoMS = Peace of Mind Scale; PHQ-9 = depression scale from the Patient Health Questionnaire; GAD-7 = Generalized Anxiety Disorder Scale. **p < 0.01 *p < 0.05.

### Multilevel Models Predicting Dream Affect from Waking Well-Being Measures

Across all measurement occasions, on average 0.25 (*SD* = 0.57) positive affects and 0.48 (*SD* = 0.78) negative affects were expressed in a dream report. Participants’ self-ratings revealed that the mean score for positive affect in a dream was 1.02 (*SD* = 0.98) and the mean score for negative affect in a dream was 0.69 (*SD* = 0.73).

**Figure 1:**
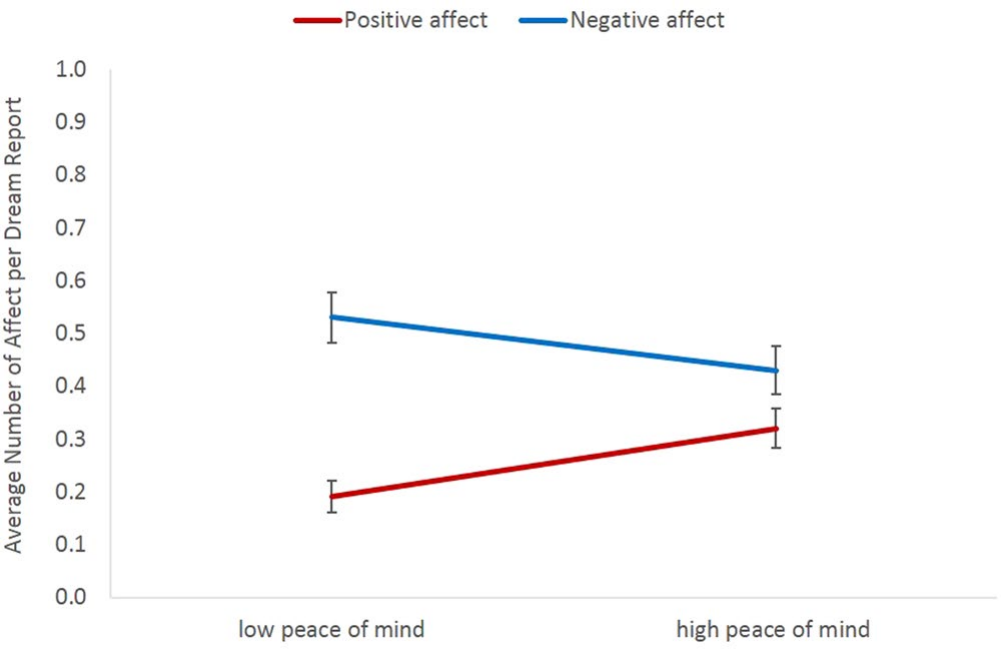
Average number of externally rated positive and negative affect per dream report in people with low (below median) vs high (above median) levels of peace of mind. The median split was used for illustrative purposes only. Error bars display standard errors of the mean.

**Figure 2:**
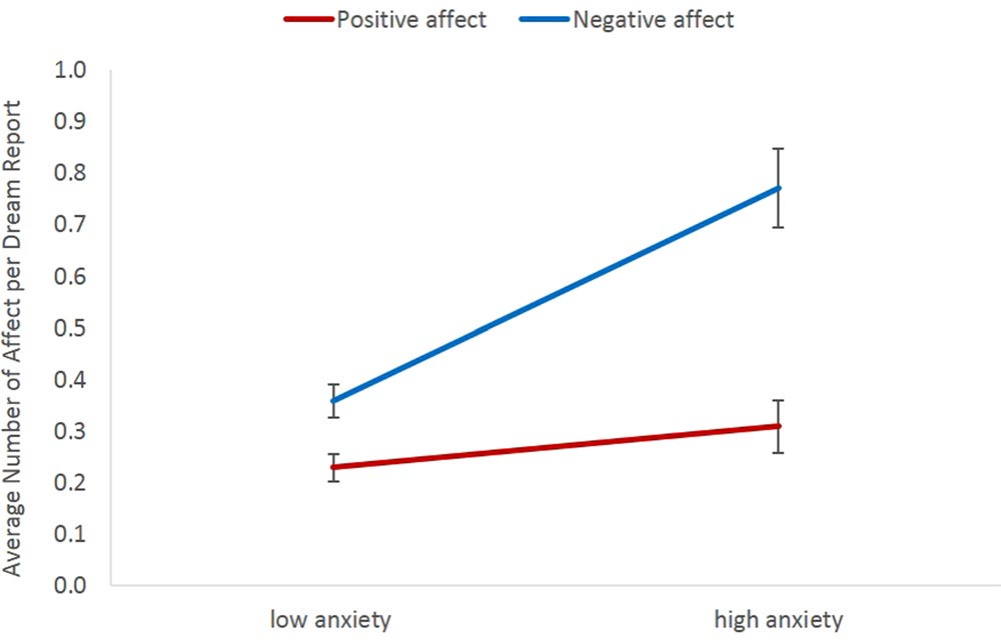
Average number of externally rated positive and negative affect per dream report in people with low (<5) vs high (≥5) levels of anxiety (GAD-7). The median split was used for illustrative purposes only. Error bars display standard errors of the mean.

Results of multilevel models regarding the relationship between waking well-being measures and dream affect are displayed in Tables 3–6. As can be seen, when controlling for all the other variables, PoMS was the only measure to predict positive affect in dream reports, whereas the scale measuring symptoms of anxiety (GAD-7) was the only measure to predict negative affect in dream reports. Regarding self-ratings of dream affect, when controlling for all the other variables, only the scale measuring symptoms of anxiety (GAD-7) predicted ratings of negative affect in dreams. For illustrative purposes only and to visualize the main findings, average number of positive and negative affect per dream report are displayed for participants divided into groups of low vs high PoM using median split on PoMS (see Fig. 1), and into groups of low vs high anxiety using a cut-off value of 5 (because this value is used to distinguish between no vs mild or more severe anxiety) on GAD-7 (see Fig. 2).

**Table 3:** Generalized Linear Mixed-Effects Model With Negative Binomial Distribution for Externally Rated Positive Affect in Dream Reports.

	*β*	*SE*	*z*	*p*	95% CI	
					*LL*	*UL*
Intercept	−2.230	0.240	−9.286	<0.001***	−2.701	−1.759
Control variables						
Female (Level-2)	0.243	0.262	0.930	0.352	−0.269	0.756
Age (Level-2)	0.386	0.109	3.540	<0.001***	0.173	0.600
Word count (Level-1)	0.003	0.001	4.574	<0.001***	0.002	0.005
Between-subject predictors (Level-2)						
Life satisfaction (SWLS)	−0.074	0.190	−0.391	0.696	−0.446	0.298
Domain satisfaction (BBQ)	0.131	0.192	0.681	0.496	−0.246	0.508
Positive affect (PA)	−0.178	0.126	−1.409	0.159	−0.426	0.070
Negative affect (NA)	0.203	0.172	1.176	0.239	−0.135	0.540
Eudaimonic well-being (FS)	−0.190	0.183	−1.035	0.301	−0.549	0.170
**Peace of Mind (PoMS)**	**0.405**	**0.155**	**2.620**	0.009**	**0.102**	**0.709**
Depression (PHQ-9)	−0.311	0.205	−1.516	0.129	−0.713	0.091
Anxiety (GAD-7)	0.248	0.181	1.366	0.172	−0.108	0.603

*Note*. All predictors, except word count and gender, were standardized. *SE* = standard error. Based on 552 observations in 44 participants. *p < 0.05. **p < 0.01. ***p < 0.001.

**Table 4:** Generalized Linear Mixed-Effects Model With Poisson Distribution for Externally Rated Negative Affect in Dream Reports.

	*β*	*SE*	*z*	*p*	95% CI	
					*LL*	*UL*
Intercept	−1.952	0.175	−11.145	<0.001***	−2.309	−1.621
Control variables						
Female (Level-2)	0.630	0.183	3.450	<0.001***	0.279	0.995
Age (Level-2)	0.297	0.078	3.792	<0.001***	0.142	0.449
Word count (Level-1)	0.004	0.000	10.229	<0.001***	0.003	0.005
Between-subject predictors (Level-2)						
Life satisfaction (SWLS)	−0.056	0.114	−0.487	0.626	−0.277	0.170
Domain satisfaction (BBQ)	0.096	0.129	0.739	0.460	−0.160	0.349
Positive affect (PA)	−0.042	0.083	−0.510	0.610	−0.203	0.123
Negative affect (NA)	−0.012	0.117	−0.106	0.916	−0.244	0.214
Eudaimonic well-being (FS)	−0.082	0.121	−0.674	0.500	−0.320	0.157
Peace of Mind (PoMS)	0.008	0.100	0.085	0.933	−0.187	0.206
Depression (PHQ-9)	−0.113	0.126	−0.903	0.366	−0.365	0.128
**Anxiety (GAD-7)**	**0.427**	**0.123**	**3.458**	<0.001***	**0.188**	**0.673**

*Note*. All predictors, except word count and gender, were standardized. *SE* = standard error. Based on 552 observations in 44 participants. *p < 0.05. **p < 0.01. ***p < 0.001.

**Table 5:** Linear Mixed-Effects Model for Self-Rated Positive Affect in Dreams.

	*β*	*SE*	*t*	*p*	95% CI	
					*LL*	*UL*
Intercept	0.902	0.143	6.317	<0.001***	0.622	1.182
Control variables						
Female (Level-2)	0.055	0.182	0.305	0.762	−0.301	0.412
Age (Level-2)	0.082	0.084	0.974	0.335	−0.083	0.246
Word count (Level-1)	0.006	0.000	1.547	0.122	−0.000	0.001
Between-subject predictors (Level-2)						
Life satisfaction (SWLS)	0.161	0.131	1.229	0.224	−0.096	0.418
Domain satisfaction (BBQ)	−0.061	0.141	−0.434	0.667	−0.337	0.215
Positive affect (PA)	0.155	0.096	1.611	0.114	−0.030	0.340
Negative affect (NA)	0.153	0.134	1.137	0.261	−0.110	0.415
Eudaimonic well-being (FS)	0.009	0.142	0.060	0.952	−0.269	0.286
Peace of Mind (PoMS)	0.099	0.117	0.846	0.402	−0.130	0.327
Depression (PHQ-9)	−0.086	0.134	−0.646	0.521	−0.349	0.176
Anxiety (GAD-7)	0.059	0.134	0.441	0.662	−0.204	0.323

*Note*. All predictors, except word count and gender, were standardized. *SE* = standard error. Based on 549 observations in 44 participants. *p < 0.05. **p < 0.01. ***p < 0.001.

**Table 6:** Linear Mixed-Effects Model for Self-Rated Negative Affect in Dreams.

	*β*	*SE*	*t*	*p*	95% CI	
					*LL*	*UL*
Intercept	0.484	0.062	7.813	<0.001***	0.363	0.605
Control variables						
Female (Level-2)	0.014	0.078	0.182	0.857	−0.139	0.167
Age (Level-2)	−0.065	0.036	−1.797	0.079	−0.137	0.006
Word count (Level-1)	0.001	0.000	8.380	<0.001***	0.001	0.002
Between-subject predictors (Level-2)						
Life satisfaction (SWLS)	0.019	0.057	0.333	0.741	−0.093	0.131
Domain satisfaction (BBQ)	0.032	0.061	0.526	0.601	−0.088	0.152
Positive affect (PA)	0.072	0.041	1.763	0.084	−0.008	0.152
Negative affect (NA)	0.033	0.058	0.568	0.573	−0.081	0.146
Eudaimonic well-being (FS)	0.015	0.062	0.237	0.814	−0.106	0.135
Peace of Mind (PoMS)	−0.015	0.051	−0.292	0.772	−0.113	0.084
Depression (PHQ-9)	0.041	0.058	0.701	0.487	−0.073	0.154
**Anxiety (GAD-7)**	**0.122**	**0.058**	**2.094**	0.041*	**0.008**	**0.236**

*Note*. All predictors, except word count and gender, were standardized. *SE* = standard error. Based on 547 observations in 44 participants. *p < 0.05. **p < 0.01. ***p < 0.001.

## Discussion

We explored the relationship between waking mental well-being and dream affect by measuring not only symptoms of ill-being but also different types and components of well-being. Moreover, this is the first time PoM was investigated as a distinct aspect of well-being in a Western sample and in relation to dream content. The results showed that when controlling for other types and components of well-being and ill-being, individuals with more PoM expressed more positive affect in subsequent dream reports. In fact, of all the measures used, PoM was a unique predictor of positive dream affect. Of ill-being measures, only anxiety was related to dream affect. Specifically, individuals with more symptoms of anxiety expressed more negative affect in subsequent dream reports and rated their dreams to contain more negative affect. The findings regarding anxiety are in line with a number of previous studies in both clinical^37,39^ and non-clinical populations^45,46^. Thus, the results demonstrate that aspects of waking ill-being and well-being are differently related to dream affect with anxiety related to negative dream affect, but peace of mind related to positive dream affect. This implies that it is not sufficient to measure only the symptoms of psychopathology, but well-being should be measured in its own right.

The finding that anxiety and PoM were related to dream affect raises the question as to what connects these aspects of ill-being and well-being, and why precisely these relate to dream affect. Anxiety is characterized by excessive anticipatory fear and chronic worry that people find difficult to control^60^. From an evolutionary perspective anxiety is a functional response to situations that are threatening or potentially threatening. In fact, it is adaptive to have a rather low threshold for detecting cues of potential danger because from the survival perspective false positives (detecting threats when they are absent) are less costly than false negatives (not detecting threats when they are present)^61^. Moreover, in an environment where threats are more common it is adaptive to have a lower threshold for threat detection. However, problems arise when the threat-detection system is chronically overactivated in a safe environment, that is, when organisms are unable to downregulate their anxiety. Thus, in relatively safe environments — which our current day environments typically are — anxiety reflects maladaptive self-regulation, specifically affect dysregulation^62^. This is supported by a panoply of neuroimaging studies which have consistently demonstrated altered function in a network of brain regions involved in the processing and regulation of affective states. Specifically, structural and functional interactions between the amygdala and regions in the medial prefrontal cortex (mPFC) and anterior cingulate cortex (ACC) have been suggested to underlie the dysregulation of affect in both clinical and non-clinical anxiety^63^. These regions are also activated in rapid eye movement (REM) sleep — the sleep stage most associated with dreaming — even to a higher extent than during wakefulness^64^. It has been suggested that the same psychological and neurobiological mechanisms underlying anxiety in wakefulness are responsible for increased negative affectivity in dreams^37^. This is consistent with, and predicted by, the simulation theories of dreaming, especially the threat simulation theory, according to which perceived waking threats (increased anxiety during wakefulness) lead to increased threat simulation (and negative affect) in dreams^9^.

PoM is characterized by inner peace and harmony that can be achieved by accepting both positive and negative experiences, balancing between positive and negative affective states, and controlling one’s mental states^17,29^. As such, enhanced self-regulation seems to underlie PoM. A study conducted on a Chinese sample provides preliminary support for this assertion^65^. Moreover, the goal of various psychotherapeutic (e.g., cognitive-behavioural therapy) and mindfulness-based (e.g., mindfulness meditation) interventions is to improve self-regulation, especially affect regulation^66–68^. Interestingly, a few studies on Chinese samples have demonstrated that those higher on trait mindfulness also report more PoM^69^ and that mindfulness training may enhance inner peace^70^. Importantly, enhanced affect regulation resulting from psychotherapeutic interventions and mindfulness practices has been shown to involve alterations in the same mPFC/ACC-amygdala circuitry that underlies anxiety^71,72^.

Based on the above, we propose that people with more PoM have enhanced self-regulation capacity in general and affect-regulation capacity in particular. Given the evolutionary adaptive function of negative affect, people with more PoM do experience negative affect (e.g., fear) but may have a higher threshold for threat detection (either due to environmental or personality factors) and be better able to regulate these states, especially when there is no imminent danger. In fact, they may upregulate positive affect as a way to cope with negative experiences^73^. Because implicit affect regulation (i.e., automatic process that does not require insight or awareness) relies on the network of brain areas activated in REM sleep (i.e., ventromedial PFC and ventral ACC)^74^, people with more PoM may display enhanced affect regulation not only in the waking state but also during dreaming. As a result, they may have more positive affect in dreams. Interestingly, lucid dreams (i.e., dreams in which one is aware of dreaming) contain more positive affect than non-lucid dreams^75^ and it has been suggested that the ability to have lucid dreams is associated with enhanced capacity for affect regulation in the waking state (and the underlying ventromedial PFC activity)^76^. Thus, we argue that whereas high levels of anxiety are characterized by relatively more negative affective states in both waking and dreaming, reflecting possible underlying affect dysregulation; high levels of PoM are characterized by relatively more positive affective states across different states of consciousness, reflecting efficient or adaptive affect regulation. Future research is needed to empirically test this theoretical proposition.

Regarding the lack of relationship between dream affect and other aspects of ill-being and well-being, it may be that these rely on at least partly distinct psychological and neurobiological mechanisms. For example, although anxiety and depression are often comorbid and both may involve dysfunctions in affect processing, there is evidence that they differ in implicit affect regulation processes^77^. Moreover, fear (rather than sadness) and worry (rather than rumination) are specifically characteristic to dream experiences according to theories which argue that the function of dreaming is fear extinction^37^ or preparation for future threats^9^. Similarly, PoM may differ from other types and components of well-being, especially with regard to self-and affective regulation processes. Although in the present study PoM was more strongly correlated with cognitive components of HWB and EWB, rather than with affective components of HWB, it is arguably conceptually distinct from all the current concepts of well-being. One can accept life’s circumstances even when these do not satisfy one’s aspirations (cognitive components of HWB), remain calm and content despite fear or sadness (affective components of HWB), and have inner peace without necessarily striving for excellence (EWB)^29^.

Alternatively, methodology may partially explain the lack of associations. In the current study waking state affect was measured only at one point in time (as part of the waking well-being questionnaire). It may well be that if positive and negative affect would have been measured on a daily basis, the results would have been in line with previous studies^51,52,54^. Also, eudaimonic well-being was measured using the Flourishing Scale^78^. Given that there is no single agreed conceptualization and operationalization of EWB, the use of different operationalizations may have resulted in different findings.

The results of the current study demonstrate that waking measures are better related to dream affect expressed in dream reports rather than self-ratings by participants. This methodological difference can thus explain, at least partly, inconsistencies in research literature. It may be that in narrative dream reports individuals selectively express only the most salient experiences, and these may differ depending on one’s level of well-being and ill-being. This is compatible with an evolutionary perspective: if affective reactions are evolutionary adaptations that increase the ability to respond appropriately in important situations, then dream reports probably explicitly describe all such most important situations and also affective responses to those events. Because we cannot directly access dream experiences, it remains an open question whether individuals with certain levels of anxiety and PoM actually experience their dreams differently or whether they simply describe or report their dream experiences differently. Nevertheless, specifically the content of dream reports may contain useful markers of both psychopathology^79^ and well-being.

The finding that the affective content of dream reports reflects certain aspects of waking ill-being and well-being is in line with the simulation theories of dreaming according to which dreams simulate waking life^80^. However, based on the current study it is not possible to say whether this content simply reflects waking well-being^6,7^ or whether it serves some specific functions in itself, such as rehearsing particular skills^80^ or (re-)processing emotions so as to function better in waking life^10–14^. Although the role of REM sleep for waking affect regulation has gained support^4,81^, the possible function of dream affect remains largely to be answered in future longitudinal and experimental studies.

Moreover, it remains to be investigated how other types of dream contents (e.g., social interactions) are related to both dream affect and waking well-being. For example, based on a recently proposed Social Simulation Theory^82^, dreaming is specialized for simulating social skills and bonds so as to strengthen social relationships in the waking life. Given the bulk of evidence that the quantity and quality of our waking social relationships is important for our well-being^83^, it would be interesting to explore whether individuals whose dream reports contain more frequent prosocial behaviours also exhibit higher waking well-being, specifically PoM.

In sum, results of the present study showed that PoM was related to positive dream affect, whereas anxiety was related to negative dream affect. Waking measures were better related to affect expressed in dream reports than to participants’ own ratings of their affective dream experiences. This is an exploratory study and more investigations with larger and more diverse sample sizes are needed to confirm these findings. Nevertheless, the study demonstrates that dream affect may reflect not only certain aspects of waking ill-being but also waking well-being, and as such, possibly serve as diagnostic or prognostic indicator of mental health in general. However, whether waking well-being influences dream affect or dream affect influences subsequent waking well-being are questions that require studies specifically designed to examine such causal relationships. Importantly, the study shows that PoM complements existing conceptualizations and measures of well-being and may help develop a more comprehensive understanding of well-being and its associations with other variables. Therefore, this aspect of well-being should be systematically integrated into the conceptual and empirical framework of well-being.

## Method

### Participants and Procedure

Data were collected as part of a larger study^58^. Participants were required to be native Swedish speakers, healthy, and to not have any psychiatric or neurological diagnoses. Forty-seven nonpaid participants volunteered for the study. After signing the informed consent, they filled in an online well-being questionnaire. Then, during the subsequent three weeks (i.e., 21 days), participants logged onto and filled in an online home dream diary every morning upon awakening in which they reported all their dreams and rated their affective experiences in those dreams. Three participants (1 man, 2 women) were excluded from the analyses because they provided less than five dream reports. Thus, the final sample consisted of 44 participants (16 men, 28 women, *M*_age_ = 26.93, *SD*_age_ = 5.09, range = 19–40 years). The study was performed in accordance with the Declaration of Helsinki and was approved by the Regional Ethical Review Board in Gothenburg, Sweden.

### Well-Being Questionnaire

The online well-being questionnaire included scales measuring all the different components of well-being, symptoms of ill-being, and sociodemographic questions. All the scales were administered in Swedish. Scales that were not available in Swedish, were translated from English to Swedish using the back-translation method^84^. One bilingual translator translated the scales into Swedish and another independent bilingual translator back-translated the scales into English. The back-translated versions of the scales were compared to the original ones by the first author of the study to ensure conceptual equivalence. Table 1 summarizes all the scales included in the well-being questionnaire and provides data about their reliability in the current sample.

#### **Life satisfaction**

The Satisfaction With Life Scale (SWLS)^85^ was used to measure how participants evaluate their life on the whole. The scale consists of five items (e.g., “I am satisfied with my life”) that are rated on a scale from 1 (strongly disagree) to 7 (strongly agree). The total score is calculated by summing up all the five items with higher scores indicating higher satisfaction with life. This scale was used because it is the most widely used scale for measuring life satisfaction and displays good psychometric properties with Cronbach’s alpha (*α*) ranging from 0.79 to 0.89^86,87^. A psychometric evaluation of the Swedish version of the scale in a nationwide sample of university students showed good reliability (*α* = 0.88) and provided support for the unidimensional model^88^.

#### Domain satisfaction

The Brunnsviken Brief Quality of Life Scale (BBQ)^89^ was used to measure how satisfied participants are with different domains of life. The scale consists of 12 items covering six life domains (leisure time, view on life, creativity, learning, friends and friendship, view of self). Participants rate the importance (e.g., “My leisure time is important for my quality of life”) and satisfaction (e.g., “I am satisfied with my leisure time: I have the opportunity to do what I want in order to relax and enjoy myself”) with each domain on a scale from 0 (do not agree at all) to 4 (agree completely). To obtain a total score, the satisfaction and importance rating for each domain are first multiplied and then the six products are summed up. This scale was used because it is a brief, open access scale validated in both clinical and non-clinical samples and has demonstrated good psychometric properties in a Swedish sample (*α* = 0.76)^89^.

#### Positive and Negative Affect

The two affective components of HWB were measured with three different scales. The Positive and Negative Affect Schedule (PANAS)^90^ was used because this is one of the most well-validated and frequently used scales to measure affect in psychological science with adequate psychometric properties^91^. It consists of 20 items with 10 items measuring positive affect (e.g., interested, proud, inspired) and 10 items measuring negative affect (e.g., distressed, hostile, ashamed). Participants were asked to rate to what extent they experienced each of the feelings during the past 24 hours on a scale from 1 (very slightly or not at all) to 5 (extremely). The positive and negative affect items were summed up to obtain the positive affect subscale (PANAS_PA) and the negative affect subscale (PANAS_NA), respectively. The Swedish versions of the subscales have shown good reliability (*α*_PA_ ranging from 0.82 to 0.87 and *α*_NE_ ranging from 0.85 to 0.86)^92,93^.

The modified Differential Emotions Scale (mDES)^55^ was included to measure a broader range of affect. The scale consists of 20 affect categories, 10 for measuring positive affect (e.g., amusement, gratitude, love) and 10 for measuring negative affect (e.g., scorn, disgust, hate). Each category is described by three adjectives (e.g., grateful, appreciative, or thankful) and participants rated the greatest amount they experienced each of those during the past 24 hours on a scale from 0 (“I did not experience any of these feelings at all”) to 4 (“I experienced one or more of these feelings extremely much”). The positive affect subscale (mDES_PA) and the negative affect subscale (mDES_NA) were obtained by calculating the mean score of the 10 positive affect categories and of the 10 negative affect categories, respectively. In previous studies the scales have demonstrated good reliability (*α*_PA_ ranging from 0.93 to 0.94 and for *α*_NA_ ranging from 0.85 to 0.86)^94,95^.

Similarly to Lee *et al*.^17^, additional items were added to ensure that both high-and low-arousal (positive and negative) affect were measured and to enable investigation of correlations between low-arousal positive affect and PoMS. Therefore, items from the 12-Point Affect Circumplex Scales (12-PAC)^96^ were included to represent the four different quadrants of the affect circumplex: high-arousal positive affect (12-PAC_HPA; excited, enthusiastic, energetic, elated), low-arousal positive affect 12-PAC_LPA; (calm, tranquil, serene, relaxed), high-arousal negative affect (12-PAC_HNA; nervous, fearful, upset, anxious), and low-arousal negative affect (12-PAC_LNA; sad, down, drowsy, tired). Participants rated to what extent they experienced each of these feelings during the past 24 h on a scale from 1 (not at all) to 5 (extremely). Mean scores for each subscale were calculated. In Lee *et al*.^17^ similar subscales demonstrated acceptable reliability (*α*_HPA_= 0.80, *α*_LPA_ = 0.80, *α*_HNA_ = 0.85, *α*_LNA_ = 0.67).

#### **Eudaimonic Well-Being**

Of the several existing scales measuring EWB, the Flourishing Scale (FS)^78^ was used because rather than addressing a specific conceptualization of EWB, it combines different conceptualizations and was specifically created to complement SWB. The scale consists of 8 items and measures important facets of optimal functioning considered central to EWB, including purpose and meaning (e.g., “I lead a purposeful and meaningful life”). Participants rate to what extent they agree with each item on a scale from 1 (strongly disagree) to 7 (strongly agree). The total score is calculated by adding up the responses to each item with higher scores indicating more optimal functioning. The scale has been shown to be unidimensional and display good psychometric properties (*α* ranging from 0.87 to 0.89)^97^. Good reliability of the Swedish version of the scale has also been demonstrated (*α* = 0.87)^98^.

#### **Peace of Mind**

The Peace of Mind Scale (PoMS)^17^ was used to measure how often participants experience inner peace and harmony in their daily life. The original scale consists of 7 items (e.g., “I have peace and harmony in my mind”) of which two items are reverse-scored (e.g., “It is difficult for me to feel settled”). The items are rated on a scale from 1 (not at all) to 5 (all of the time) and the mean of the item scores reflects an overall measure of peace of mind. Although PoMS was originally developed to measure well-being in the Chinese culture, Lee *et al*.^17^ demonstrated it to be a valid and reliable measure also in a Western sample (European Americans). However, because in the US sample the two reverse-coded items loaded on a separate factor, the authors excluded these two items and used the five-item PoMS instead (*α* = 0.90). We assessed the factor structure of the Swedish version of the scale in a pilot sample of 140 Swedish university students (54 men, 86 women; *M*_age_ = 25.30, *SD*_age_ = 6.72, range = 19–59 years). Confirmatory factor analysis (using the lavaan function in R^99^) indicated the two-factor solution to be a better fit (*χ*^2^ = 21.70, *df* = 13, *p* = 0.060, RMSEA = 0.07, CFI = 0.98, SRMR = 0.03, AIC = 2505.24) than the one-factor solution (*χ*^2^ = 27.57, *df* = 14, *p* = 0.016, RMSEA = 0.08, CFI = 0.97, SRMR = 0.04, AIC = 2509.15). Therefore, we used the five-item PoMS in subsequent analyses. However, to enable comparison with Lee *et al*.^17^, we present the reliability, descriptive statistics, and correlations with other well-being measures for both the five- and seven-item PoMS. We also repeated the multilevel analyses using the seven-item PoMS and the results were essentially the same as those obtained using the five-item PoMS.

#### **Depression**

The depression module of the Patient Health Questionnaire (PHQ-9)^100^ was used to measure symptoms of depression. The brief 9-item scale is based on the diagnostic criteria from the Diagnostic and Statistical Manual of Mental Disorders (DSM-5)^101^ and used in both research and clinical practice. Participants rate how often, over the last two weeks, each of the symptoms (e.g., “Little interest or pleasure in doing things”) has bothered them on a scale from 0 (not at all) to 3 (nearly every day). The total score is obtained by summing up the ratings for each item and the cut-off scores of 5, 10, 15, and 20 represent mild, moderate, moderately severe and severe depression, respectively. It is a widely used open access depression screening instrument demonstrating good psychometric properties (*α* = 0.87) in the general (i.e., non-clinical) population^102^.

#### **Anxiety**

The Generalized Anxiety Disorder Scale (GAD-7)^103^ was used to measure symptoms of anxiety. The 7-item scale is based on the most prominent features of the diagnostic criteria A, B, and C for generalized anxiety disorder from the DSM-5^101^. Participants rate how often, over the last two weeks, each of the symptoms (e.g., “Worrying too much about different things”) has bothered them on a scale from 0 (not at all) to 3 (nearly every day). The scores for each item are summed to obtain a total score with 5, 10, and 15 representing mild, moderate, and severe anxiety, respectively. The scale has shown good psychometric properties (*α* = 0.89) in the general (i.e., non-clinical) population^104^.

#### **Additional measures**

In addition to mental well-being, participants also answered questions about their physical well-being, physical ill-being, and subjective sleep quality. Because the current study focused on mental well-being, these data were not included in the analyses.

### Home Dream Diary

Participants were asked to write down their dreams every morning upon awakening during a three-week period. To counteract forgetting, participants were instructed to jot down their dreams (using pen and paper) immediately upon awakening, but before getting up. Thereafter, they were to log onto and fill in an online daily dream diary. In this diary participants reported whether they remembered having any dreams that night and, in case they did, provided a detailed description of the dream. Specifically, they were asked to write down the dream in as much detail as they could remember (what happened, where, when, who was present, what they felt and thought). Participants were asked to report their dreams as completely and truthfully as possible, and to not edit, censor, interpret or elaborate the dream reports beyond what they remembered happening. If participants wished to comment on some aspects of the dream, they were asked to add their comments in brackets or at the end of the dream reports so that these would be clearly distinguishable from the actual dream (report). Following Antrobus^105^, the length or word count of each dream report was calculated by counting together all dream-related words, excluding fillers, repetition, corrections, and waking comments. After reporting the dream, participants rated the feelings they experienced in the dream using the mDES^55^. If participants remembered several dreams from the same night, they were asked to report the dream and rate the dream affect separately for each dream. In case participants had not logged onto the online dream diary on some day(s), an email reminder was sent.

#### **Self-ratings of dream affect**

Self-ratings of dream affect were obtained using the mDES scale. This scale was used to ensure comparability and consistency with our previous study^59^. Participants were asked to rate the extent to which they experienced each of the 20 affect categories in their dream on a scale from 0 (“I did not experience any of these feelings at all”) to 4 (“I experienced one or more of these feelings extremely much”). Similarly to the waking affect ratings, the mean scores of the 10 positive and 10 negative affect categories were calculated to obtain the positive affect subscale (Dreams_SR_PA) and the negative affect subscale (Dreams_SR_NA), respectively (min = 0; max = 4) (see Supplementary Materials for examples of dream reports and self-ratings of dream affect).

#### **External ratings of dream affect**

All dream reports were combined, randomized, and all identifying information removed. Two judges content analysed the reports according to the criteria, procedure, and measures used in Sikka *et al*.^59^. First, the judges worked independently and identified each and every occasion when affect was explicitly expressed in the dream report (as experienced by the dream self in the dream), could be unambiguously inferred from the behaviour of the dream self, or both. However, because the interrater percent agreement was low for cases where affect was inferred (53.7%), subsequent analyses included only the 503 cases where affect had been explicitly expressed. The interrater percent agreement for explicitly expressed affect was 84.7% and for cases where affect was both expressed and inferred 73.9%. Then, the judges independently classified these occurrences of affect using the mDES. In addition to the 20 mDES categories an additional “Other” category was used for cases that were difficult to classify into the existing categories (altogether 8.3% occurrences of affect). Interrater reliability using Cohen’s kappa^106^ indicated almost perfect agreement between the judges’ classification of affect (*κ* = 0.92). The judges did not rate the intensity of affect because the reliability and validity of such ratings is questionable^6^. The mDES categories that had at least one occurrence per dream report were counted together to form a positive affect (Dreams_ER_PA) and negative affect (Dreams_ER_NA) subscale (min = 0; max = 10) (see Supplementary Materials for examples of dream reports and external ratings of dream affect). For a more detailed description of the content analysis process and interrater reliability calculations, see Sikka *et al*.^58^.

### Statistical Analyses

Because measurement occasions (i.e., ratings of daily dream affect, *N* = 552, *M* = 12.55, *SD* = 5.72) were nested within individuals (*N* = 44), data were analysed using multilevel regression models, also known as mixed model analysis or hierarchical linear modelling^107^ in the R statistical program (Version 3.4.1 R Development Core Team, 2017). These models allow for dependency across measurement occasions and can deal with unbalanced designs in which different individuals have a different number of measurement occasions. Moreover, these models allow for between- and within-person variation simultaneously, which results in more precise estimation of standard errors of regression coefficients. In addition, the modelling of the within-person variation generalizes the results to the population mean level, in terms of the fixed effects. In the present study, we focused on fixed terms describing the mean response in the population level.

There were four separate outcome variables representing the ratings of dream affect with self- and external ratings: (1) external ratings of positive affect (Dreams_ER_PA); (2) external ratings of negative affect (Dreams_ER_NA); (3) self-ratings of positive affect (Dreams_SR_PA); (4) self-ratings of negative affect (Dreams_SR_NA). The first two outcome variables (1–2) represent count data (i.e., the number of times different categories of affect were rated to occur in the dream report). Count data are often modelled with Poisson regression. However, if the data show more variation than expected, Type-I errors may result. Because preliminary analyses showed a marked overdispersion (i.e., observed variance was larger than the mean) in the outcome variable Dreams_ER_PA, we used generalized linear mixed-effects models for the negative binomial family (glmer.nb function in R^108^). The outcome variable Dreams_ER_NA was not overdispersed and thus the generalized linear mixed-effects model with poisson distribution and log function was used (glm function in R^108^). In both of these models there was subject-specific random mean-response included in the model. The other two outcome variables (Dreams_SR_PA and Dreams_SR_NA) represent continuous data (i.e., mean scores of dream affect ratings) and therefore linear mixed-effects models with maximum likelihood estimation (using lmer function in R^108^) were used. There was a subject-specific random intercept included in the model. Additionally, because the residuals for the model including the outcome variable Dreams_SR_NA were not normally distributed, this outcome variable was square-root transformed before the model was fit.

Thus, a series of two-level regression models were fitted in which the dream affect ratings were Level-1 outcome variables and the scores on well-being scales Level-2 predictor variables. Because the four positive and four negative waking affect scales aimed to measure the same underlying construct and also displayed very high correlations among each other (> 0.8), a composite measure for waking positive affect and negative affect was created. For this, the affect scales were first recoded to be on the same scale (i.e., 1–5) and then a mean of all the four different scales (e.g., mean of PANAS_PA, mDES_PA, 12-PAC_HPA and 12-PAC_LPA) calculated. This was done separately for the positive and negative affect yielding a composite positive affect scale (PA) and a composite negative affect scale (NA). As such, there were in total eight Level-2 predictor variables (SWLS, BBQ, PA, NA, FS, PoMS, PHQ9, GAD7). Multicollinearity — the degree to which the predictors are correlated — was evaluated using multicollinearity diagnostics. The variance inflation factor (VIF) and tolerance are the most commonly used methods for detecting multicollinearity. VIF values exceeding 5 or 10 and tolerance values remaining below 0.1 or 0.2 are usually considered problematic^109–111^. The multicollinearity diagnostics (calculated using the vif function in the usdm package^112^) showed that for all predictors VIF remained below 4 (except for the FS for which VIF = 4.28) and tolerance above 0.2 which indicates sufficient independence among the predictors. Removing the predictor with the highest VIF (i.e., FS) and repeating all the analyses did not alter the results.

All Level-2 predictor variables were standardized and grand mean centered. In all the analyses we controlled for the length of each dream report to account for the fact that ratings of affect are more likely to occur in longer dream descriptions. Therefore, the length of the dream report (word count) was entered as a Level-1 predictor in the analyses. Additionally, to account for gender differences, gender was controlled for and dummy-coded variables (0 for men; 1 for women) were entered as Level-2 predictors in the analyses. Also, age was controlled by adding the standardized and grand mean centered age as a Level-2 predictor in the models.

Because all the well-being variables were standardized and centered before the analysis, the estimated coefficients indicate a magnitude of change in the outcome variable associated with an increase of one standard deviation in the predictor variable.

## Electronic supplementary material

Dataset 1

## Data Availability

The datasets generated during and/or analysed during the current study are available from the corresponding author on reasonable request.
