# Supplementary material for: Peace of mind and anxiety in the waking state are related to the affective content of dreams
Source: Sci Rep. 2018 Aug 24;8:12762. doi: 10.1038/s41598-018-30721-1 (PMC6109051; doi:10.1038/s41598-018-30721-1)
Supplement: Supplementary file 1 — Dataset 1 [file 41598_2018_30721_MOESM1_ESM.docx]

**Electronic Supplementary Material for**

**Peace of Mind and Anxiety in the Waking State Are Related to the Affective Content of Dreams**

Pilleriin Sikka ^1 2 *^, Henri Pesonen ^3 4^, Antti Revonsuo ^1 2^

^1^ Department of Psychology and Speech-Language Pathology, Turku Brain and Mind Center, University of Turku, Finland

^2^ Department of Cognitive Neuroscience and Philosophy, School of Bioscience, University of Skövde, Sweden

^3^ Department of Mathematics and Statistics, University of Turku, Finland

^4^ Department of Computer Science, Aalto University, Finland

^*^ Correspondence concerning this article should be addressed to:

Pilleriin Sikka

Department of Psychology and Speech-Language Pathology

Turku Brain and Mind Center

University of Turku

Finland

Email: [pilsik@utu.fi](mailto:pilsik@utu.fi); [pilleriin.sikka@his.se](mailto:pilleriin.sikka@his.se)

**Examples of Dream Reports**

Below are examples of dream reports (translated from Swedish) from two participants: one participant with low levels of peace of mind (PoM) and high levels of anxiety, and another with high levels of PoM and low levels of anxiety. The respective scores on the Peace of Mind Scale (PoMS) and the Generalized Anxiety Disorder Scale (GAD-7) are presented in parentheses. External ratings of dream affect (i.e., dream affect as expressed in dream reports) and self-ratings of dream affect (i.e., dream affect as rated by participants) are presented on the right with ratings for positive affect (PA) and negative affect (NA) detailed separately. Dream report contents in parentheses refer to waking commentary that was excluded from word count. To ensure the anonymity of participants, place names have been removed and replaced with XX.

|  | Number of positive and negative affects expressed in a dream report  (min = 0, max =10) | | Mean score of 10 positive and 10 negative affects in a dream as rated by participants  (min=0, max=4) | |
| --- | --- | --- | --- | --- |
|  | PA | NA | PA | NA |
| **Female; Low PoM (PoMS: 1.80); High Anxiety (GAD-7: 11)** |  |  |  |  |
| I was at a party with my boyfriend and many of his friends. We were in a house which I do not know where or whose it was. My boyfriend got really drunk early in the evening and danced very silly and I thought he made a fool of himself. Afterwards I wanted to talk to him, but he was upset because I thought he was embarrassing. Then *I got angry* and went out and smoked in protest (I do not smoke, and my boyfriend does). Then he also got angry because he thought I was stupid. *I was mostly amused*. Suddenly, we were together again and were on the way home to his parents’ place who live in XX. Then my boyfriend suddenly wanted to go to XX, there was a ferry leaving from his parents' pier and it went very fast across to XX, it felt like 15 minutes. There we bought fishing lures and compared each other's fishing lures and their colour. (Then I woke up.) | 1 | 1 | 1.30 | 0.50 |
| I was on a big bus in the mountains. There was a snow storm and we were heading to a hotel high on the mountain (it did not look like a high mountain). The bus driver was very difficult to communicate with and I and a friend of mine needed to pee. I do not remember who the friend was. In the end we arrived at the hotel and I would sit and pee in the woods next to where the bus was parking. We were going to go back on the bus, but then the bus driver said he could not wait a minute longer and then he left. Once again, *I was very annoyed* at the bus driver. There were some other people at the bus stop who were going take the next bus that was supposed to arrive any minute, but it had been delayed for 40 minutes so I didn’t really believe it would arrive. A puppy was there too (I cannot remember why. Then I woke up). | 0 | 1 | 0.80 | 0.70 |
| I was in a big prison, there were me, Leonardo DiCaprio and several prisoners. There was something weird happening in the whole building and we found out that every time someone left the room and closed the door behind him, a copy of that person was created somewhere. This meant that we never knew if it was the "real" person who came back or not. We realized we had to hold the door/doors as soon as someone went out, then wait for him to come back (so that the door would not close as usual). The elevators were especially difficult because the doors were closing by themselves. It was *very stressful* every time someone had to leave the elevator and then come back to us in it. We used a piece of gravel to secure the door. But some did not make it back and thus got copied. I could also see the room in the prison where all the copies ended up being. I don’t know how they ended up there. (The whole dream felt very much like an ongoing movie, like Inception. Then I woke up.) | 0 | 1 | 1.30 | 0.67 |
| I was together with a bunch of people and was picking vegetables. There was some sort of competition where we had to look for something or pick the most vegetables. We were divided into teams. Somebody suddenly found a sheep’s brain which was *very disgusting*. I was wearing very nice high boots. (The rest is very vague.) | 0 | 1 | 0.40 | 0.70 |
| I suddenly realized that I had an extra apartment near the University of XX, around XX. I had lived there for a while but forgotten about it and stayed with my boyfriend in another apartment (the one we live in now). We had been looking for a smaller one so I went there to check it out. I looked at the keychain, even though there were only four keys in the bunch of keys, I did not find the door key. I was *very frustrated*. I only found a key that matched the mailbox so I opened the mailbox and found lots of candies that I had put there in an earlier occasion. *I was happy* and picked up the candy, then tried to use the key to open the door of the apartment and it worked. There was a lot of construction work and construction workers outside the door. I entered the apartment and found lots of old shoes that both me and my boyfriend had forgotten there and that we had been looking for. I cleaned the apartment and ate the candy. (Then I woke up.) | 1 | 1 | 2.10 | 0.40 |
| I went to Liseberg (amusement park) with an old classmate and his girlfriend from XX. We went to Balder (a roller coaster) and another roller coaster. We also ate ice cream and I did not bring the ice cream with me to the roller coaster but put it on a table. My classmate’s girlfriend had never been to Liseberg before, but she loved it. | 0 | 0 | 1.60 | 0 |
| **Male; High PoM (PoMS: 4.40); Low Anxiety (GAD-7: 0)** |  |  |  |  |
| I attended a lecture which I had the feeling was given in XX or XX near my hometown. I went into the lecture hall and sat down on a free seat and next to me was one of the coaches of the Swedish national team in football. I sat down and after a while we started talking a little. Then I meet them both in the locker room. I'm on my way to the shower and they're getting out of there and we decide they should come to my hometown because they've never been there and would love to see it*. I felt happy* and thought it was *amazing*. It was an old building I was in, I was standing in the shower and suddenly started *to feel a little bit uncomfortable.* (Then I woke up.) | 2 | 1 | 3.80 | 0.60 |
| I stepped into a hot air balloon and went high, high up. Underneath me everything was so beautiful with different colours, very strong colours and it was like in a fairytale world. I could jump from the air balloon and yet land very softly in the trees where the leaves were like giant cotton pads. I did it and *it was a lovely feeling*. I did it when the balloon had started descending so that I would not lose the balloon. I was in an old little house with great hospitality and where other balloon travelers came to meet. Then it was time to go on and I went to my balloon in the beautiful landscape. | 1 | 0 | 4 | 0.20 |
| I was sitting at a table in the living room of my childhood home and it felt like I was about 15 years old. In front of me on the table there were lots of ornaments. My dad was lying on the floor behind me. I wanted to get down from the table, but if I had moved, I would have certainly wiped some of the ornaments off the table. In the end I decided to try to turn and turn around to get in a good position to get down and when I did this I moved some of the ornaments and it became a domino effect and everything on the table crashed down from the table. My dad got angry and started yelling at me and *I in turn screamed out of anger* with every muscle in my body tense. A moment later my parents were leaving and hugging my siblings. My dad came to me and had calmed down and when he approached me, I said: “do not touch me”. | 0 | 1 | 0 | 2.50 |
| I was back at my old workplace after having finished my studies and when I went there for the first time, I had to enter from the back side, considering the whole place was being renovated and all the activities were taking place in the barracks. The teachers came to the back and welcomed me and everything seemed to be a mess and the teachers themselves seemed confused. Then there were many faces I did not recognize either. But *I was still glad* to be back there. | 1 | 0 | 3.40 | 0.20 |
| I was playing in a basketball match and in this game the atmosphere between the teams was very tense and a lot of audience was standing. But at one point during the match, the other team receives an incorrect call and they also lose points because of this. So when I take the ball to their side of the field, I choose to put the ball on the floor so they can take it and then I run home to defend. They take advantage of the situation and score in this attack. Then my team does the same thing; an incorrect call is made against us, the opponents put the ball on the floor which is to our advantage. I feel that it's very even between the teams, I get the ball and jump to the basket and miss the first and the second time, then a teammate comes and throws the ball into the basket. The crowd gets crazy and we win the match. | 0 | 0 | 2.90 | 0.40 |
| I was in a city I don’t know and something *scary* had taken place, so we had to get away from there. I sat on top of a high fence and people were standing below this and one person and another one was climbing towards me and I helped them over the barbed wire and then over a ladder that was on my left side. There was a child on her way and she did not dare to get over the fence and after some attempts, her mother said she should come down again so they could try to find another way out. | 0 | 1 | 1.00 | 0.40 |
